# Association of *MME* gene polymorphisms with susceptibility to Alzheimer's disease in an Iranian population

**DOI:** 10.1016/j.heliyon.2024.e37556

**Published:** 2024-09-06

**Authors:** Fereshteh Khalili-Moghadam, Javad Hosseini Nejad, Taleb Badri, Morteza Sadeghi, Javad Gharechahi

**Affiliations:** a Student Research Committee, Baqiyatallah University of Medical Sciences, Tehran, Iran; b Neuroscience Research Center, Baqiyatallah University of Medical Sciences, Tehran, Iran; c Human Genetics Research Center, Baqiyatallah University of Medical Sciences, Tehran, Iran

**Keywords:** Alzheimer's disease, SNP polymorphism, *MME*, Neprilysin

## Abstract

**Background:**

the *MME* gene encodes a membrane metalloendopeptidase, known as neprilysin (NEP). There are no reports on the potential implications of *MME* gene polymorphisms on the risk of Alzheimer's disease (AD) in the Iranian population. In this study, we studied the potential association of two single nucleotide polymorphisms (SNPs), rs6797911 and rs3736187, in the *MME* gene and the risk of developing AD in an Iranian population.

**Methods:**

This case-control study comprised 120 AD-diagnosed patients and 120 healthy individuals without any prior family history of AD. The patient and control groups were matched for major demographic and health characteristics. Genotyping was performed by amplification refractory mutation system-polymerase chain reaction (ARMS-PCR).

**Results:**

All patients included in this study were assessed by an experienced neurologist to exclude cases with other forms of dementia based on a brain computed tomography scan and other clinical findings. There were no significant differences in demographic and health characteristics including sex, diabetes, blood pressure, and cigarette smoking status between case and control groups (p > 0.05). However, the age difference appeared significant. Both SNPs were significantly associated with the risk of AD in our study population. The rs3736187 (T > C, 3:155168489) was strongly associated with AD risk under the log-additive model (OR = 1.67, CI = 1.18–2.37, p-value = 0.003). The rs6797911 (T > A, 3:155144601) also showed a significant association with AD risk under the dominant model (TT vs. TA and AA, OR = 3.37, CI = 1.86–6.1, p-value <0.001).

**Conclusion:**

There is a strong association between *MME* gene polymorphisms and susceptibility to AD in the Iranian population. Amyloid-β (Aβ) can serve as a substrate for the NEP metalloendopeptidase, the product of the *MME* gene. However, the mechanistic understanding of how these genetic variations affect NEP expression, function, and consequently susceptibility to AD, is poorly understood. Further research is required to fully understand the exact implication of *MME* gene variations on AD, particularly in a larger, ethnicity-diverse population.

## 1 Introduction

Alzheimer's disease (AD) is a common neurodegenerative disease characterized by dementia, progressive decline in cognitive functions, and loss of the ability to perform basic daily living activities [1]. Early diagnosis and intervention are crucial, as unchecked disease progression can become life-threatening, and the existing therapies are most effective in the early stages. AD is characterized by the formation of senile plaques and the neurofibrillary tangles in the brain cortex which is followed by inflammatory responses and neuronal death [2,3]. These plaques and tangles are mainly formed by the extracellular deposition of amyloid-β (Aβ) peptides and the intraneuronal accumulation of misfolded hyperphosphorylated microtubule-associated protein tau, respectively [4,5]. The formation of senile plaques and neurofibrillary tangles in the brain is believed to begin long before the manifestation of clinical symptoms in the patient. Although plaque formation is a hallmark of AD, the exact mechanisms of disease development and progression remain elusive. Studies have shown that the formation of Aβ plaques and neurofibrillary tangles disrupt neuronal function and communication between neurons [6]. Over time, these pathological changes lead to neuronal dysfunction, synaptic and neuronal loss, and brain atrophy, ultimately leading to cognitive decline and the progression of AD.

AD is a genetic disorder primarily affecting older adults, with a heritability rate ranging from 60 to 80 % [7]. While mutations in *APP*, *PSEN1*, and *PSEN2* genes are linked to the familial early-onset form of AD [[8], [9], [10]], genome-wide association studies (GWAS) have pinpointed over 80 genes associated with the more common late-onset form of AD [3,[11], [12], [13]]. The ε4 allele of the *APOE* gene is the strongest genetic risk factor for sporadic late-onset AD, accounting for about 4 % of the genetic variations [14]. Significant contributions to AD development also come from genetic variations in *SORL1*, *TREM2*, and *ABCA7* genes [15]. However, these common variants account for only a small fraction of the disease's heritability [13,16]. Despite these discoveries, many cases of AD cannot be fully explained by currently known genetic variations, suggesting that additional factors or undisclosed genetic loci may play a role. This underscores the necessity for further research with larger and more diverse sample populations to uncover additional risk loci. Such studies should also consider the complex interplay between genetic factors and environmental influences, such as lifestyle and diet, which contribute to the complexity of AD.

Emerging GWAS and functional analysis data suggest that pathways associated with Aβ production and clearance play a critical role in AD pathogenesis [17,18]. Notably, evidence indicates that early-onset familial AD is characterized by increased Aβ production [19], whereas late-onset AD is linked to impaired Aβ clearance [20]. Efforts have been made to develop therapeutics targeting soluble and insoluble Aβ or the enzymes responsible for amyloid precursor protein (APP) cleavage as potential AD treatments, albeit with disappointing outcomes [21]. Among these enzymes, neprilysin (NEP) stands out as a key player in Aβ degradation within the brain [22]. NEP is a type II membrane metallopeptidase that targets many small peptide substrates including Aβ, substance P, angiotensin I, angiotensin II, enkephalins, bradykinin, oxytocin, and neurotensin [[23], [24], [25]]. Studies have consistently shown that NEP activity declines with age, leading to increased Aβ accumulation [26,27]. Furthermore, research on postmortem brain samples from AD patients confirms decreased NEP expression and activity [28,29]. While the role of NEP in Aβ clearance is widely acknowledged, studies in AD mouse models have shown that NEP overexpression reduced soluble Aβ monomer levels but failed to affect pathogenic oligomers, resulting in no improvement in cognitive functions [30].

NEP is encoded by the *MME* gene. Variations in the *MME* gene can affect NEP expression and activity, potentially influencing AD pathogenesis. Notably, the SNP rs9827586 within the *MME* gene has shown significant associations with both NEP protein levels and enzymatic activity [28]. Additionally, a study investigating 22 polymorphisms in the *MME* gene among AD patients highlighted a robust association between the rs1836915 polymorphism and AD susceptibility [31]. A previous genetic association study in a large case-control cohort of the Han Chinese population also suggested a strong association of the SNP rs1816558 in the *MME* gene with AD risk, even after adjustment for the ε4 allele of the *APOE* gene [32]. This indicates that NEP is an important enzyme contributing to genetic susceptibility to AD. There is currently no report on the association of *MME* gene polymorphisms and AD susceptibility in the Iranian population, which is largely distinct from the Han Chinese or other study populations. In this study, we investigated the association between two common SNPs (rs3736187 and rs6797911) within the *MME* gene with AD risk in a case-control population from Iran.

## 2 Material and methods

### 2.1 Patient selection and blood sampling

In this case-control study, we included 240 participants, comprising 120 AD patients and 120 healthy control individuals. Participants were recruited from Baqiyatallah Hospital Clinic between July 2022 and June 2023. AD patients were identified based on clinical examinations conducted according to the criteria outlined by the National Institute of Neurological and Communicative Disorders and Stroke/Alzheimer's Disease and Related Disorders Association (NINCDS-ADRDA). The following exclusion criteria were considered for AD participants: recent alcohol or drug dependence within the past six months, documented brain injury or drug use in recent medical records, inherent cognitive impairment, mental illnesses, or other forms of cognitive decline, serious physical illnesses, and organic brain disorders. Control subjects were selected from age-matched individuals who exhibited no signs of abnormalities or dementia. Table 1 shows the demographic and health characteristics of the study population. Each participant received detailed information about the research objective and provided informed consent through a consent form that adhered fully to the ethical standards outlined in the Declaration of Helsinki. Approval for this study was obtained from the Ethics Committee of Baqiyatallah Hospital (IR.BMSU.BAQ.REC.1401.134).

**Table 1:** Demographic and health characteristics of the study population.

Group		Control (healthy)	Case (Alzheimer's disease, AD)	p-value	95 % CI
Age ± SD (year)		58.76 ± 10.85	66.96 ± 9.89	3.81e-09	−10.85 − −5.56
Gender	male	75	65	0.24	0.81 − 2.44
	female	45	55		
Smoking status	+	20	25	0.5	0.37 − 1.53
	–	100	95		
Diabetes status	+	10	13	0.66	0.28 − 1.94
	–	110	107		
Blood pressure	+	4	6	0.75	0.13 − 2.85
	–	116	114		

### 2.2 DNA extraction and genotyping

**Fig. 1:**
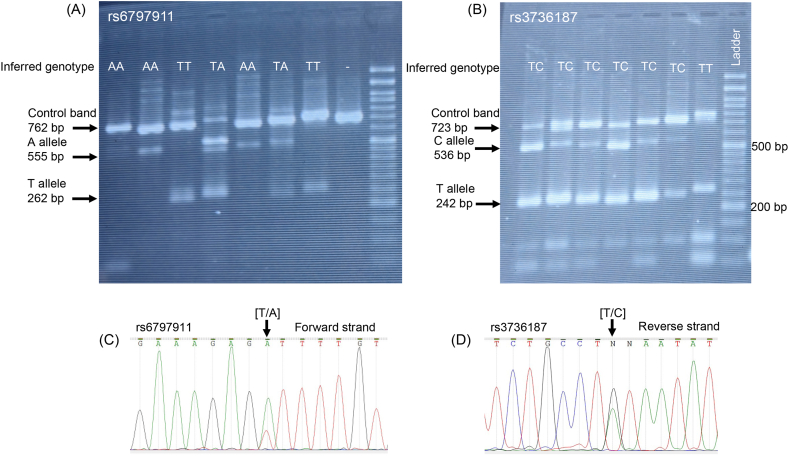
SNP genotyping using tetra-primer amplification refractory mutation system (TETRA-ARMS). Agarose gel electrophoresis results of eight randomly selected samples (both case and control) genotyped for rs6797911 (A) and seven samples genotyped for rs3736187 (B). Sanger sequencing chromatogram of a heterozygous individual for rs6797911 (C) and rs3736187 (D). The SNP position is shown using a vertical arrow.

A 3 mL peripheral blood sample was collected from each participant. Genomic DNA extraction was carried out manually using the salting-out method. The quality of the extracted DNA was assessed using both Nanodrop spectrophotometry and gel electrophoresis. The genotyping of candidate SNPs was conducted using the tetra-primer amplification refractory mutation system-polymerase chain reaction (Tetra-ARMS PCR) method. The primers used for genotyping of candidate SNPs are detailed in Table 2. PCR was performed in a 25 μL reaction, consisting of 12.5 μL of 2X PCR red master mix (Amplicon), 30 ng of template genomic DNA, 0.2 μL of each outer primer (from a 50 pmol stock solution), and 1.4 μL of each inner primer (from a 50 pmol stock solution). The PCR conditions consisted of 1 cycle of denaturation at 94 °C for 4 min, followed by 35 cycles of denaturation at 94 °C for 40 s, annealing at 57 °C for 30 s, and extension at 72 °C for 25 s, with a final extension cycle at 72 °C for 5 min. The PCR products were then electrophoresed on a 2 % agarose gel along with a 50 bp DNA size marker, and the bands were visualized using a gel documentation instrument (Fig. 1A and B). To validate the accuracy of our genotyping method, a subset of individuals heterozygous for each SNP was subjected to Sanger sequencing (Fig. 1C and D).

**Table 2:** The sequence of primers used for tetra-primer ARMS-PCR genotyping of candidate SNPs.

Primer name	Orientation	Primer sequence	Length
rs3736187			
r187OF	Outer forward	TGTCTGGGTCAATGCTGAGAAG	22
r187OR	Outer reverse	AGTGTTCAGCTCCGTCACATC	21
r187IF	Inner forward (allele C)	CCCATTTTACTTAAATAAATATATGAC	27
r187IR	Inner reverse (allele T)	GTCTCCATCTTTGTTAAAGTTTCTGCATA	29
rs6797911			
r911OF	Outer forward	TGATTGCACAGATCCGAGAAGT	22
r911OR	Outer reverse	TCAGGGCTCTTTGAGAGCTGA	21
r911IF	Inner forward (allele A)	CAATATGTAGTGTTTCCAGAAAGTGA	26
r911IR	Inner reverse (allele T)	ATTGTAGAAATATATCTTTATCCACAATAA	30

### 2.3 Statistical analysis

All statistical analyses were conducted using R statistical software. The association between SNP polymorphisms and AD was assessed using logistic regression, implemented in the association function in the R package SNPassoc [33]. Hardy-Weinberg equilibrium (HWE) testing was performed using the tableHWE function from the SNPassoc package. Additionally, genotype frequencies were evaluated using Fisher's exact test in R. The differences in clinical and demographic features between healthy controls and AD patients were analyzed using either a Student's t-test or the Fisher's exact test in R. A p-value less than 0.05 was considered statistically significant.

## 3 Results

This study included 120 AD patients (65 males and 55 females) and 120 healthy controls (75 males and 45 females), aiming to investigate the association between candidate SNPs and the risk of AD development in a sample of the Iranian population recruited to the Baqiyatallah Clinic. The average ages for the AD and control groups were 66.96 (±9.89 years) and 58.76 (±10.85 years), respectively. The study population was not well matched for age, as significant differences in age distribution were observed between the case and control groups (*t*-test p-value <0.001). Differences in other patient demographic or health characteristics, including sex, blood pressure, smoking status, and diabetes status were not significant (Table 1). Testing for HWE in both case and control groups yielded nonsignificant results for rs6797911 (p-value = 0.09). However, rs3736187 deviated significantly from HWE (p-value = 1.3e-06).

**Fig. 2:**
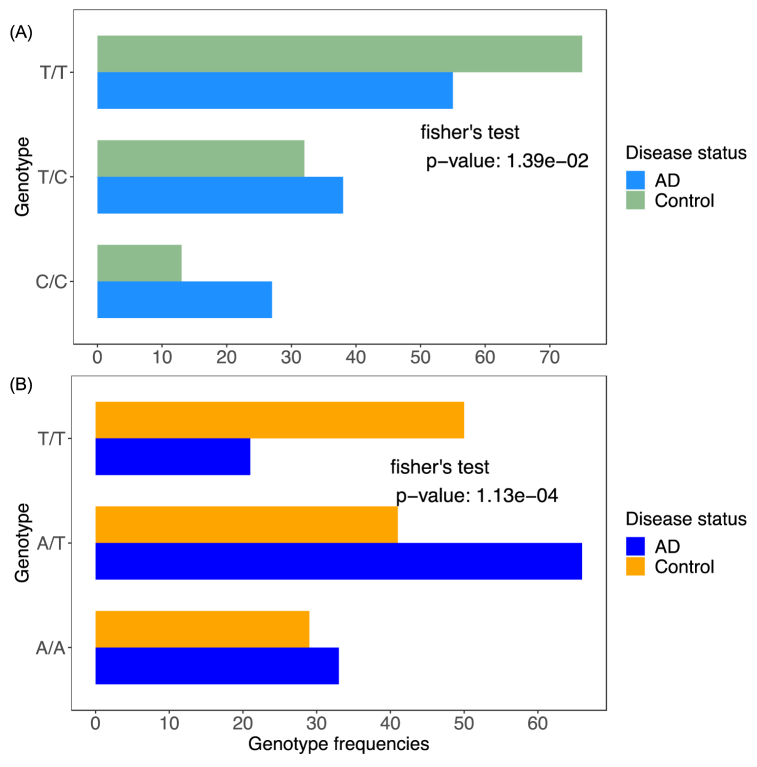
SNPs genotype distribution in case and control groups. Genotype frequencies for rs3736187 (A) and rs6797911 (B) in AD and control groups. The differences in genotype frequencies were tested using Fisher's exact test. P-value <0.05 is considered statistically significant.

Fig. 2 shows the genotype distribution for the two candidate SNPs in the AD and control groups. For both SNPs, there were significant differences in genotype distributions between the study groups (Fisher's exact test, p-value <0.05). In both polymorphisms, the genotypes containing alternate alleles were more abundant in AD group compared to the control group. Table 3 presents the association analysis for the two SNPs under various modes of inheritance. Notably, SNP rs3736187 demonstrated a significant association across all inheritance models, except for the overdominance model (p-value <0.05). The most significant result was obtained under the log-additive model with an OR = 1.67, CI = 1.18–2.37, and a p-value = 0.003 with the lowest Akaike information criterion (AIC) of 328.1. Stratifying for age and sex did not affect the association results. Although age differences were significant between the case and control groups, including age as a covariate in the regression analysis did not significantly alter the association results. Allele association analysis further indicated a robust association of the C allele with the AD status for this variant (OR = 1.95, p-value = 0.01), consistent with the genotype association findings.

**Table 3:** Association analysis of rs3736187 and rs6797911 SNPs in the *MME* gene in AD patients and healthy individuals under different modes of inheritance. AD: Alzheimer's disease, OR: odds ratio, AIC: Akaike information criterion, CI: 95 % confidence interval.

rs3736187 (T > C)									
Mode of inheritance	Genotype	No. control	%	No. AD	%	p-value	OR	AIC	95 % CI
Co-dominant	TT	75	62.5	55	45.8	0.0135	1.00	330.1	–
	TC	32	26.7	38	31.7		3.83		0.90–2.91
	CC	13	10.8	27	22.5		2.71		1.34–5.98
Dominant	TT	75	62.5	55	45.8	0.0093	1.00	330.0	–
	TC-CC	45	37.5	65	54.2		3.37		1.18–3.30
Recessive	TT-TC	107	89.2	93	77.5	0.0144	1.00	330.7	–
	CC	13	10.8	27	22.5		1.19		1.17–4.90
Over-dominant	TT-CC	88	73.3	82	68.3	0.393	1.00	336.0	–
	TC	32	26.7	38	31.7		2.36		0.73–2.23
Log-additive	0,1,2	120	50.0	120	50.0	0.00337	1.67	328.1	1.18–2.37
rs6797911 (T > A)									
Co-dominant	TT	50	41.7	21	17.5	0.00103	1.00	320.4	–
	AT	41	34.2	66	55.0		3.83		2.02 − 7.28
	AA	29	24.2	33	27.5		2.71		1.33 − 5.53
Dominant	TT	50	41.7	21	17.5	0.000033	1.00	319.5	–
	TA-AA	70	58.3	99	82.5		3.37		1.86 − 6.10
Recessive	TT-TA	91	75.8	87	72.5	0.552	1.00	336.4	–
	AA	29	24.2	33	27.5		1.19		0.67 − 2.12
Over-dominant	TT-AA	79	65.8	54	45.0	0.0011	1.00	326.1	–
	TA	41	34.2	66	55.0		2.36		1.40 − 3.97
Log-additive	0,1,2	120	50.0	120	50.0	0.00397	1.66	328.4	1.17 − 2.36

Association analysis for the rs6797911 variant revealed a robust association under the dominant model, with an OR of 3.37, CI of 1.86–6.10, and a p-value of 0.00003, accompanied by a low AIC value of 319.5 (Table 3). Similar to the rs3736187 variant, a significant association was also observed under the log-additive model (OR = 1.66, CI = 1.17–2.36, p-value = 0.004), indicating that the presence of the alternate allele (A allele) significantly elevated the risk of AD development. When the data were stratified for age and sex, no significant deviation in association results was noted.

**Fig. 3:**
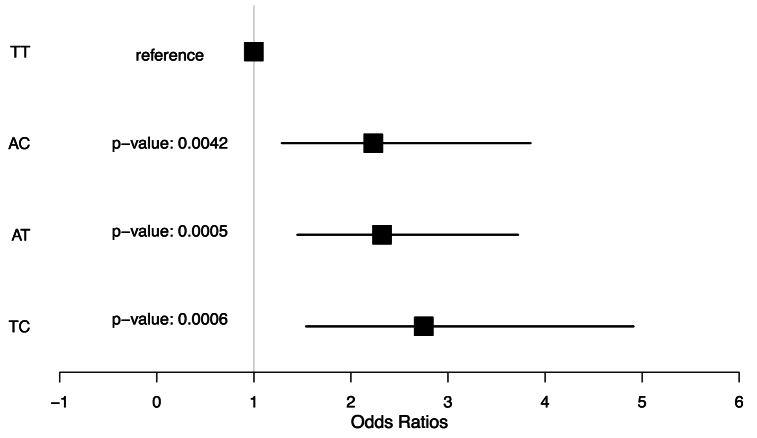
The forest plot shows the association of the four possible haplotypes of the two candidate SNPs in the MME gene with AD status. The reference was the TT haplotype, formed by the combination of both SNPs' reference alleles. Since the alternative alleles of both SNPs were significantly associated with AD, all haplotypes that contained at least one alternate allele (risk allele) also showed an association.

We also investigated the association of haplotypes formed by the two variants. Using the TT haplotype, composed of the reference alleles of both SNPs, as a reference, we observed significant associations with AD for the remaining three possible haplotypes (Fig. 3). Given that the alternate alleles in both SNPs were linked to the AD risk, all haplotypes containing at least one risk allele also exhibited an association. These data further confirm a strong association between these SNPs in the *MME* gene and the risk of AD development.

## 4 Discussion

The primary aim of this study was to investigate the association between two candidate SNPs (rs3736187 and rs6797911) within the *MME* gene and the risk of AD in an Iranian population. Our analysis revealed a significant association between these candidate SNPs and AD risk under multiple inheritance models, with the strongest signal observed under the log-additive and dominant models. In both SNPs, the presence of the alternate allele was linked to a substantially increased AD risk. Haplotype analysis further corroborated the findings, as haplotypes harboring risk alleles of either or both SNPs exhibited significant associations with AD.

Studies across diverse populations have yielded a complex picture regarding *MME* gene polymorphisms and AD risk. Associations between specific SNPs in the *MME* gene (e.g. rs989692, rs373618, rs701190, rs6797911, rs1816558, and rs6665) and susceptibility to AD have been reported in China [32,34], the United Kingdom [28], Finland [35], Spain [36], and Japan [37] populations. However, there are also reports that did not find a significant link between *MME* gene variations and susceptibility to AD [38,39]. This inconsistency suggests the potential population-specific effects or the involvement of other genetic and environmental factors that may interact with *MME* gene variations to influence AD risk. For example, sequence variations in the 3′-untranslated region (3′-UTR) of the *MME* gene have been associated with increased AD risk in an age-dependent manner [36]. In addition, in a case-control study involving the Han Chinese population, a robust association between rs1816558 in the *MME* gene and AD risk emerged only after adjusting for the ε4 allele of the *APOE* gene [32], indicating a potential gene-gene interaction in AD susceptibility. These findings indicate that the association between *MME* gene polymorphisms and AD risk is complex, varying across different populations and potentially influenced by other genomic factors.

The associations observed between *MME* gene variants and AD risk in our study population align with findings from other ethnic groups. For example, rs6797911 has been linked to AD risk in case-control populations from the United Kingdom, Italy, and Sweden [28], as well as in genome-wide association studies of late-onset AD [40,41]. Additionally, there are reports of SNPs in the *MME* gene, such as rs2016848, which are in close proxy with rs6797911 (r^2^ = 0.86), being associated with AD risk [42]. However, linkage disequilibrium (LD) analysis did not reveal any other SNPs in the *MME* gene that could serve as a proxy (r^2^ > 0.8) for rs6797911 and show potential association with AD. Nonetheless, other variants that are in strong LD with rs6797911 have been associated with different clinical conditions. For instance, rs3773885 in the *MME* gene, a close proxy (r^2^ = 0.89) for rs6797911, has been linked to type 1 diabetes [43].

Consistent with our findings, an association of rs3736187 with AD risk has been reported in case-control studies conducted in Finnish [35] and Tibetan [34] populations and in a meta-analysis [44]. However, conflicting reports exist, with some studies failing to associate this variant with an increased AD risk [28,45], indicating a population-specific pattern of association. Furthermore, LD analysis did not reveal any potential AD-linked proxy variant for this polymorphism in the *MME* gene. Given that the genotype distributions for this variant did not follow HWE, caution should be taken when interpreting the potential association observed here, as it could stem from factors such as population structure or problems with the genotyping procedure.

The *MME* gene encodes the neprilysin (NEP) enzyme, a highly conserved membrane metalloendopeptidase expressed ubiquitously in various tissues, including the brain. NEP plays a crucial role in degrading the amyloid-β (Aβ) peptide, the primary constituent of senile plaques that are hallmarks of AD pathogenesis [24]. NEP can degrade both monomeric and pathological oligomeric forms of the Aβ peptide [46]. Studies using mouse models of AD have demonstrated that the downregulation of NEP during the early stages of AD development leads to an enhanced accumulation of Aβ peptide in the brain [47,48]. Heterologous overexpression of human NEP in the mouse brain was shown to increase Aβ peptide clearance, reduce amyloid load, and improve memory function by 50 % [49]. Genetic association studies have also revealed a robust link between polymorphisms in the *MME* gene, such as rs9827586, and the expression and activity of NEP protein [28]. NEP protein expression is also reduced in the temporal and frontal cortex of the brain in normally aged and AD patients [50,51]. Loss-of-function mutations in NEP have been linked to Charcot-Marie-Tooth (CMT) disease [52,53], a neurodegenerative disorder characterized by peripheral neuronal loss and muscular atrophy. However, there is currently no clinical evidence indicating that individuals with CMT are at a higher risk of developing AD compared to the general population. This suggests that other mutations may interact with NEP mutations to predispose individuals to AD; alternatively, the absence of NEP may be compensated by other proteolytic systems.

As loss-of-function mutations in the *MME* gene are not associated with increased susceptibility to AD, variations at this locus could potentially serve as proxies for other loci that predispose individuals to AD. Previous studies have also indicated that while variations in the *MME* gene could increase AD risk, there is no evidence that this effect is mediated through changes in NEP expression or activity [28]. Both variants studied here (rs3736187 and rs6797911) are located within the intronic regions of the *MME* gene. While they may not directly impact NEP activity, they may influence gene expression, splicing, or the stability of NEP mRNA. Alternatively, these variants might be linked to other functional polymorphisms that affect NEP expression and activity, either within the *MME* locus or elsewhere. Considering these findings alongside the pivotal role of NEP in clearing Aβ peptides in the brain, it could potentially serve as a marker for measuring susceptibility to AD and as a target for drug development and therapeutic interventions.

The strengths of this study include the well-characterized case-control design, the use of robust statistical methods for genetic association analyses, and the consistency of the findings across multiple genetic models. However, some limitations should be noted. The study was conducted in a small sample of the Iranian population, and expanding the results to the general population or other ethnic groups requires further validation. Additionally, the underlying molecular mechanisms by which the identified *MME* variants influence AD risk warrant further functional investigations.

In conclusion, this study provides compelling evidence supporting the role of genetic variations within the *MME* gene as risk factors for AD in the Iranian population. These findings contribute to the growing body of literature implicating NEP-mediated Aβ clearance in the pathogenesis of AD. Further replication studies and mechanistic investigations are needed to solidify the clinical relevance of *MME* gene variants as potential biomarkers and therapeutic targets for AD.

## Informed consent statement

Informed written consent was obtained from all eligible participants. Participants who agreed to participate signed the consent form before the start of the study.

## Data availability statement

The complete dataset for this study is accessible upon request from the corresponding author.

## CRediT authorship contribution statement

**Fereshteh Khalili-Moghadam:** Writing – original draft, Visualization, Methodology, Investigation, Data curation. **Javad Hosseini Nejad:** Resources, Data curation. **Taleb Badri:** Validation, Resources. **Morteza Sadeghi:** Resources, Data curation. **Javad Gharechahi:** Writing – review & editing, Supervision, Project administration, Data curation, Conceptualization.

## Declaration of competing interest

The authors declare that they have no known competing financial interests or personal relationships that could have appeared to influence the work reported in this paper.
